# Elevated Circulating Microparticle Subpopulations in Incidental Cerebral White Matter Hyperintensities: A Multimodal Study

**DOI:** 10.3390/brainsci11020133

**Published:** 2021-01-20

**Authors:** Che Mohd Nasril Che Mohd Nassir, Mazira Mohamad Ghazali, Amanina Ahmad Safri, Usman Jaffer, Wan Zaidah Abdullah, Nur Suhaila Idris, Mustapha Muzaimi

**Affiliations:** 1 Department of Neurosciences, School of Medical Sciences, Universiti Sains Malaysia, Kubang Kerian 16150, Kelantan, Malaysia; nasrilche123@gmail.com (C.M.N.C.M.N.); mazira.mohamadghazali@gmail.com (M.M.G.); amaninaahmadsafri@gmail.com (A.A.S.); jafferu@gmail.com (U.J.); 2 Department of Haematology, School of Medical Sciences, Universiti Sains Malaysia, Kubang Kerian 16150, Kelantan, Malaysia; wzaidah@usm.my; 3 Hospital Universiti Sains Malaysia, Jalan Raja Perempuan Zainab II, Kubang Kerian 16150, Kelantan, Malaysia; nursuhaila@usm.my; 4 Department of Family Medicine, School of Medical Sciences, Universiti Sains Malaysia, Kubang Kerian 16150, Kelantan, Malaysia

**Keywords:** cerebral small vessel disease, cardiocerebrovascular, microparticles, diffusion MRI

## Abstract

Asymptomatic (or “silent”) manifestations of cerebral small vessel disease (CSVD) are widely recognized through incidental findings of white matter hyperintensities (WMHs) as a result of magnetic resonance imaging (MRI). This study aims to examine the potential associations of surrogate markers for the evaluation of white matter integrity in CSVD among asymptomatic individuals through a battery of profiling involving QRISK2 cardiocerebrovascular risk prediction, neuroimaging, neurocognitive evaluation, and microparticles (MPs) titers. Sixty asymptomatic subjects (mean age: 39.83 ± 11.50 years) with low to moderate QRISK2 scores were recruited and underwent neurocognitive evaluation for memory and cognitive performance, peripheral venous blood collection for enumeration of selected MPs subpopulations, and 3T MRI brain scan with specific diffusion MRI (dMRI) sequences inclusive of diffusion tensor imaging (DTI). WMHs were detected in 20 subjects (33%). Older subjects (mean age: 46.00 ± 12.00 years) had higher WMHs prevalence, associated with higher QRISK2 score and reduced processing speed. They also had significantly higher mean percentage of platelet (CD62P)- and leukocyte (CD62L)-derived MPs. No association was found between reduced white matter integrity—especially at the left superior longitudinal fasciculus (LSLF)—with age and neurocognitive function; however, LSLF was associated with higher QRISK2 score, total MPs, and CD62L- and endothelial cell-derived MPs (CD146). Therefore, this study establishes these multimodal associations as potential surrogate markers for “silent” CSVD manifestations in the well-characterized cardiocerebrovascular demographic of relatively young, neurologically asymptomatic adults. Furthermore, to the best of our knowledge, this study is the first to exhibit elevated MP counts in asymptomatic CSVD (i.e., CD62P and CD62L), which warrants further delineation.

## 1. Introduction

Cerebral small vessel disease (CSVD) consists of a spectrum of histopathological, clinical, and imaging abnormalities linked to pathology in the microvasculature of small penetrating arteries and arterioles (50–500 µm) in brain irrigating subcortical structures [1,2,3]. Generally, it results in brain parenchyma injuries, which are invariably associated with distal leptomeningeal and intracerebral vessel pathology, affecting deep cerebral white matter integrity [4]. Current data suggest that CSVD is one of the most prevalent neurological disorders in aging societies of the developed world [5,6]. The prevalence of apparently asymptomatic manifestations of CSVD, such as silent brain infarcts or white matter hyperintensities (WMHs), has been associated with advancing age [7].

“Silent” or “asymptomatic” manifestations of CSVD are frequently detected as an incidental finding from brain imaging and are often used as a prognosis marker following a first symptomatic CSVD presentation [8]. In magnetic resonance imaging (MRI), the micro-changes in white matter can be seen as WMHs that represent more diffuse areas of white matter lesions [9]. Such changes, as seen on a conventional MRI, have shown limited correlation with clinical parameters (e.g., cognition), ranging from weak to moderate [10]. Thus, more concise correlations and reliable lesion surrogates are required in order to enhance the evaluation of white matter structure integrity, including white matter tractography. Advancements in diffusion MRI (dMRI) techniques have offered potential new information on disease pathomechanisms as well as surrogate markers for disease onset and progression, and new therapy evaluations using newer magnetic resonance (MR)-based diffusion tensor imaging (DTI) modalities [11,12].

Furthermore, as part of risk evaluation, essential clinical algorithms have become useful in the prevention and management of CSVD, including cerebrovascular events [13]. In this line, QRISK has been developed to evaluate cardiovascular risk as well as the risk of cerebrovascular diseases such as CSVD. QRISK is an online-based tool that predicts cardiocerebrovascular disease risk over the next 10 years [14]. However, the correlation with MR-DTI findings in white matter changes using QRISK and its neurocognitive evaluations performance among asymptomatic individuals are still insufficient. Moreover, at present, systematic data on CSVD are nonexistent in Malaysia.

Microparticles (MPs) are non-nucleated, small (0.1–1 μm in diameter), and membrane-enclosed extracellular particles [15,16,17], which are formed through various triggered mechanisms—including cellular activation, injury, or apoptosis—which cause membrane phospholipids exocytic blebs to be released from the cell surface [18,19,20]. MPs compositions are heterogeneous and can originate from different cell types, such as platelets, leukocytes, erythrocytes, and endothelial cells [21,22]. They inherit their parental cell surface proteins and cytoplasmic materials [23]. Their formation and accumulation have been reported to affect the proper functioning of small vessels. A previous study has shown that endothelial dysfunction caused by MPs can induce vascular inflammation, potentially leading to a prothrombotic state in arteriosclerosis and atherosclerosis [22]. Furthermore, MPs shedding has been implicated in ischemic stroke subtypes [24]. In CSVD, such a manifestation is related to the disorganization of arterial segmental walls and luminal narrowing, where the accumulation of MPs (alongside cholesterol crystals) may trigger hypoperfusion, leading to microinfarcts and WMHs [25,26].

Furthermore, variations in MPs and their subpopulation counts in various diseases have indicated their diagnostic importance, particularly in vascular pathologies. However, most of the previous studies regarding MPs have been skewed toward disease populations and did not focus on asymptomatic subjects with a prevalence of WMHs. Hence, in the present study, we sought to find novel correlations and associations of MPs and their subpopulation counts with an imaging modality, QRISK scores, and neurocognitive profiles as potential surrogate markers for CSVD among asymptomatic individuals with well-characterized cardiovascular demographics in suburban southeast Peninsular Malaysia.

## 2. Materials and Methods

### 2.1. Ethics Approval, Sample Size Estimation, and Subject Recruitment

The present study was conducted at the Hospital Universiti Sains Malaysia, a suburban tertiary referral center for neurological disorders on the east coast of Peninsular Malaysia, with a catchment population base of over 4 million people. Ethics approval was obtained from the Human Research Ethics Committee at Universiti Sains Malaysia (USM/JEPeM/15030096). Subjects were recruited through simple convenience random sampling (among those who attended the Family Medicine Clinic at HUSM for their routine medical follow-up appointments). Demographic (i.e., age, sex, and race) and clinical (i.e., smoking status, diabetes, body mass index, and systolic blood pressure) information were collected before cardiocerebrovascular risk calculation using QRISK2. The QRISK2 version 2018 (http://www.QRISK.org/index.php; the University of Nottingham and EMIS), an online-based cardiocerebrovascular calculator, was used to calculate the percentage of disease risk (over the next 10 years) for all recruited subjects. The projected risk for the subjects was arranged according to the following criteria: 0 to 10% = no/low cardiovascular risk; 10.1 to 20% = moderate risk; and ≥ 20.1% = high risk [13]. Asymptomatic subjects aged 25–62 years without any medical symptoms and no history of neurological diseases as well as “no/low to moderate cardiovascular risk” were included in the present study. Subjects with “high cardiocerebrovascular risk” percentage and/or with a previous history of neurological diseases were excluded.

### 2.2. Neurocognitive Assessment

The Wechsler Adult Intelligence Scale: Fourth edition (WAIS-IV) was used to evaluate the general cognitive and memory abilities of the subjects. The perceptual reasoning index (PRI) from WAIS-IV was used to calculate nonverbal reasoning and perceptual organization through three subtests: block design, matrix reasoning, and visual puzzle. The working memory index (WMI) was used to specifically measure simultaneous and sequential processing, attention, and concentration through two subtests: digit span and letter–number sequencing. Finally, the processing speed index (PSI) was used to calculate the speed of mental and graphomotor processing through two subtests: coding and symbol search. Scores obtained from each subtest were used for the multimodal comparison.

### 2.3. MPs Enumeration

A total of 18 mL of fasting peripheral venous blood samples was collected from each subject using BD Vacutainer^®^ tubes (Becton Dickinson, BD, Plymouth, UK) containing active anticoagulant trisodium citrate with citric acid and dextrose (solution B). A multi-sample Luer Adaptor (Terumo, Malaysia) and 21-gauge needles (Terumo Corporation, Biñan, Philippines) were used to aid in blood drawing. To minimize platelet activation during blood sampling, no tourniquets were used. As much as possible, MPs generation was prevented by transporting the tubes with minimal agitation and by keeping them at 25 °C, following previously published recommendations [27].

The blood samples were centrifuged at 2500× *g* for 15 min at 24 °C to separate the plasma from blood cells and platelets [28]. The platelet-poor plasma was separated through the second centrifugation step (2000× *g*, 10 min, 24 °C), in order to obtain platelet-free plasma (PFP). A third centrifugation step (2000× *g*, 10 min, 24 °C) was carried out to better separate the PFP. Enumeration of MPs was performed directly in fresh PFP in order to avoid MPs loss and to preserve MPs characteristics [27]. The unused test samples were kept in a −80 °C freezer.

Before flow cytometry analysis, 50 µL of freshly isolated PFP was incubated in the dark for 20 min at 4 °C after being diluted with 50 µL of 0.5 × Annexin-V binding buffer (Becton Dickinson (BD), Bioscience, USA), followed by staining with fluorescein isothiocyanate Annexin-V (2 µL, Becton Dickinson (BD), Bioscience, Franklin Lakes, NJ, USA) and fluorescence-conjugated monoclonal antibodies: APC CD62P (platelet-derived MPs; 2 µL, Becton Dickinson (BD), Bioscience, USA), PE-Cy 7 mouse anti-human CD235a (erythrocytes-derived MPs; 2 µL, Becton Dickinson (BD), Bioscience, USA), PE CD62L (leucocytes-derived MPs; 2 µL, Becton Dickinson (BD), Bioscience, USA), and PE-Cy7 mouse anti-human CD146 (endothelial cell-derived MPs; 2 µL, Becton Dickinson (BD), Bioscience, USA).

Thereafter, 350 μL of 1 × Annexin-V binding buffer was added to the samples. They were then shaken gently and analyzed by flow cytometry assay (FACS Canto II, Becton Dickinson, BD Biosciences, USA) at a low-rate setting. All MPs were measured using both forward scatter and side scatter. The gating mechanism was based on previous studies [18,29,30,31]. We defined MPs, for the purposes of the present study, as particles that have a diameter less than 1.0 µm. Thus, MPs that had positive staining for Annexin-V and expressed specific markers were separated as true events from background noise. The samples were run for flow analyzed at rates below 10,000 events/s and stopped when 10,000 events within the MPs gate were collected or stopped after a fixed time of 5 min. The MPs subpopulation counts were calculated as the number of events per MPs gate, whereas total MPs count was calculated by a summation of multiple MPs subpopulation counts.

### 2.4. Diffusion MRI Protocols

A Philips Achieva (Best, The Netherlands) 3T MRI machine with a 32-channel head coil (*b*-value: 1000 s/mm^2^) was used for brain scanning with the following acquisition parameters: For structural image acquisition 3D-T1, echo time (TE)/repetition time (TR) = 10/678 ms, reconstruction matrix = 512 × 512 × 40, field of view (FOV) = 230 mm, voxel size = 0.45 mm × 0.45 mm, slice spacing = 0 mm, slice thickness = 2.5 mm, flip angle = 70°, and 180 contiguous sagittal slices orientation; for T2, TE/TR = 80/3000 ms, reconstruction matrix = 512 × 512 × 24, FOV = 230 mm, voxel size = 0.45 × 0.45, slice spacing = 1.0 mm, slice thickness = 2.5 mm, and flip angle = 90°; for 3D-fluid attenuated inversion recovery (FLAIR), TE/TR = 125/11,000 ms, TI = 2800 ms, reconstruction matrix = 512 × 512 × 24, FOV = 230 mm, voxel size = 0.45 mm × 0.45 mm, slice spacing = 0 mm, slice thickness = 2.5 mm, flip angle = 90°, and 170 contiguous sagittal slices orientation; and for DTI, a scheme of 32 directions with a b-value of 1000 s/mm^2^, TE/TR = 72/6951 ms, reconstruction matrix = 96 × 96, FOV = 240 mm, voxel sizes = 2.5 mm × 2.5 mm, slice spacing = 0 mm, slice thickness = 2.5 mm, and flip angle 90°. The total scanner time was 15–20 min, per the subject’s ability.

### 2.5. Image Analysis

Images were first visualized by an assigned radiologist using the MeDINria version 2.2 software [32] in order to manually detect the presence of WMHs. The severity was evaluated using the Fazekas scale [33,34]. The presence of WMHs was further confirmed using the white matter lesion segmentation technique. This procedure was used to automatically detect and evaluate WMHs. To do this, lesion segmentation tools (LSTs) were used. The LST was evaluated in functional MRI Toolbox of Statistical Parametric Mapping version 12 (Institute of Neurology, University College London, London, UK) and installed through the Matrix Laboratory (MATLAB) version 2017a software (The MathWorks, Natick, MA, USA).

The DSI studio software (http://dsi-studio.labsolver.org) was used for image processing in white matter tractography for subjects with WMHs (WMH^+^) and those without WMHs (WMH^−^). In DSI studio, a DTI diffusion scheme was used for image reconstruction and tensor estimation. A total of 32 diffusion sampling directions were acquired. The *b*-value was 1000 s/mm^2^. The in-plane resolution was 2.5 mm. The slice thickness was 2.5 mm. The diffusion tensor was calculated.

### 2.6. Region of Interest Analysis and Tractography

With the anatomical reference from FLAIR imagery, a voxel-based region of interest (ROI) identification technique was applied over the selected WMHs in the axial plane, followed by regional tractography. A deterministic fiber tracking algorithm was used, as previously described [35]. The seeding region was placed over the whole brain. An ROI was placed at the region where the WMHs were located. The angular threshold was 60°, and the step size was 1.25 mm. The anisotropy threshold was determined automatically by DSI studio. Tracks with length less than 30 mm were discarded. A total of 5000 seeds were placed. Thereafter, the structure of the white matter tracts where the ROI was located was defined using a white matter atlas (John Hopkins University DTI-based White Matter Atlases, provided by DSI studio). Hence, the structures of the white matter tracts involved in WMHs were identified. Furthermore, the ROI for WMH^−^ subjects was guided by the white matter tracts involved in WMHs first identified in WMH^+^ subjects. The atlas of white matter tracts identified was set as a benchmark for parameter comparison between WMH^−^ and WMH^+^. Hence, the comparison between WMH^+^ and WMH^−^ was performed based on structures of the white matter tracts involved rather than on specific ROIs.

### 2.7. Statistical Data Analysis

The collected data were analyzed using the Statistical Package for Social Sciences version 24 software (IBM Corp., Armonk, NY, USA). Alpha (α) was set at 0.05, and for all analyses, *p* > 0.05 was considered statistically significant with a confidence interval of 95%. Descriptive statistical analysis, such as mean and standard deviation, was applied to all data. Correlations between variables were obtained using Pearson’s correlation coefficient. Mean differences between and within variables were obtained using an independent sample *T*-test for significant normally distributed data; meanwhile, the Mann–Whitney U test was used for nonnormally distributed data. A linear regression model was used to study the associations between age, QRISK2 score, neurocognitive function, and MPs. These analyses were adjusted for gender, ethnicity, and prior cardiocerebrovascular disease risk factors. Subsequent analyses were conducted in order to investigate the associations of DTI parameters (i.e., tract specific integrity), adjusted for age, QRISK2 score, neurocognitive function, and MPs subpopulations.

## 3. Results

### 3.1. Demographics and Cardiocerebrovascular Risk Prediction

Sixty subjects (mean age: 39.83 ± 11.50 years) who fulfilled the inclusion and exclusion criteria for the sample were recruited. The majority were female, Malay ethnicity, nonsmokers, and non-hypertensive (Table 1). Based on the QRISK2 profiles, the overall mean percentage of subjects that will develop cardiocerebrovascular risk for the next 10 years was 2.83 ± 4.42%. Furthermore, all subjects were at no/low–moderate risk, signifying that the subjects of the present study were potentially normal/healthy, with low or no chance of developing vascular diseases (i.e., asymptomatic). Older subjects (≥40 years old; mean QRISK2: 3.99 ± 3.75%) had a significantly higher QRISK2 percentage (*p* = 0.00 < 0.05) than younger subjects (≤39 years old; mean QRISK2: 0.34 ± 0.29%). Hence, the mean QRISK2 of older subjects was higher than that of the younger subjects.

### 3.2. Age vs. the Proportion of WMHs among the Study Subjects

**Figure 1:**
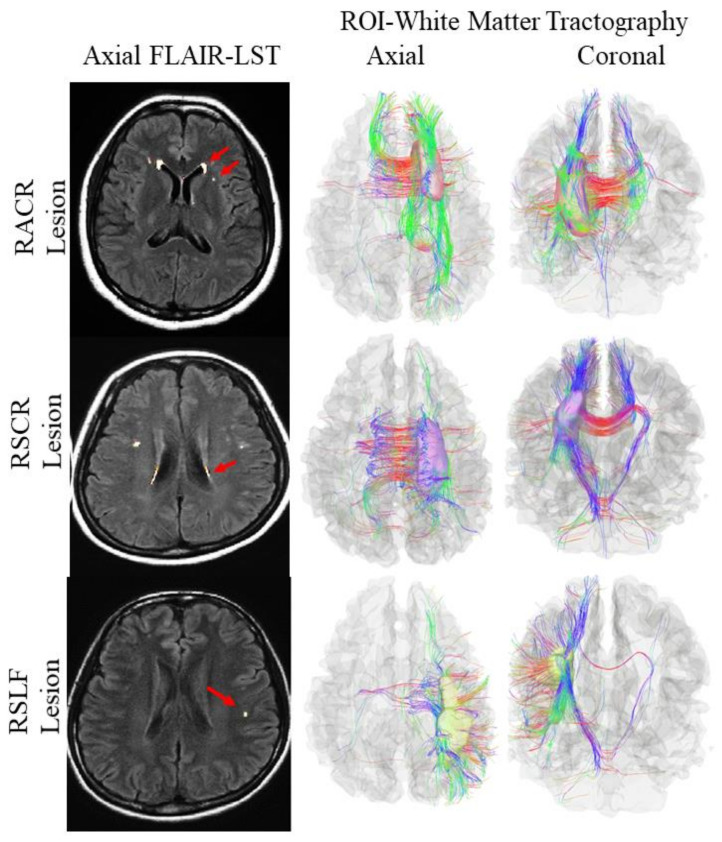
From left: the first row shows fluid attenuated inversion recovery (FLAIR) images with white matter hyperintensities (WMHs) voxel outputs from the lesion segmentation tool (LST) showing right anterior corona radiata (RACR) lesion (red arrow) and RACR tractography (axial and coronal view). The second row shows FLAIR images with WMHs voxel outputs from the LST showing right superior corona radiata (RSCR) lesion (red arrow) and RSCR tractography (axial and coronal view). The third row shows FLAIR images with WMHs voxel outputs from the LST showing right superior longitudinal fasciculus (RSLF) lesion (red arrow) and RSLF tractography (axial and coronal view). The blue, red, and green colors on the right figures represent white matter fibers running along the inferior–superior, right–left, and anterior–posterior orientations, respectively. Notes: LST followed the native Statistical Parametric Mapping (SPM) and Matrix Laboratory (MATLAB) orientations for producing JPEG images; hence, the original radiological view (left on right) obtained from the magnetic resonance imaging (MRI) scanner was shifted to neurological view (right on right) as a reminder of viewing interpretation. FLAIR, fluid-attenuated inverse recovery; RACR, right anterior corona radiata; RSLF, right superior longitudinal fasciculus; RSCR, right superior corona radiata; ROI, region of interest.

Among the 60 subjects, 20 (33%) were identified as WMH^+^ (mean age: 46.00 ± 12.00 years) with grade 1 Fazekas (see Figure 1) and the rest were identified as WMH^−^ (mean age: 36.75 ± 10.04 years). Among the WMH^+^ subjects, 14 (70%) were among the older subjects (mean age: 49.70 ± 7.95 years). There was a significant age difference between WMH^−^ and WMH^+^ subjects (see Table 2). Therefore, in the present study, WMHs were more prevalent in older individuals. Nevertheless, we also found WMHs among the younger subjects (mean age: 36.75 ± 10.04 years).

### 3.3. QRISK2 vs. the Proportion of WMHs among the Study Subjects

We found that WMH^+^ subjects had a significantly higher mean percentage of QRISK2 than WMH^−^ subjects (Table 2). In the present study, the prevalence of WMHs was higher in older subjects; in the same line, QRISK2 has a stronger correlation with age, as shown by the linear model (Table 3).

### 3.4. Neurocognitive Profiles vs. the Proportion of WMHs among the Study Subjects

We found that WMH^−^ subjects had higher mean scores of PRI, WMI, and PSI than WMH^+^ subjects, although the differences were not significant (see Table 2). Furthermore, we also found that age and QRISK2 had negative correlations with the neurocognitive performance of the study subjects. However, the linear model only revealed a significant relationship between age and PSI (Table 3). Therefore, it seems that processing speed is reduced with advancing age, especially in asymptomatic individuals with WMHs.

### 3.5. MPs vs. the Proportion of WMHs among the Study Subjects

We found that WMH^+^ subjects had higher individual MPs counts than WMH^−^ subjects. The differences were significant only for leukocyte-derived MPs (CD62L) and platelet-derived MPs (CD62P) but not for red blood cell-derived MPs (CD235a) and endothelial cell-derived MPs (CD146) (Table 2). Total MPs counts were also significantly higher in WMH^+^ subjects. We also found a weak to moderate positive correlation between the age and QRISK2 of subjects with MPs counts (Table 3). The strongest correlation was between CD62L with age and QRISK2. However, the linear model showed that CD62L was significantly associated with QRISK2 only, not with age. Furthermore, no significant correlation was found between MPs counts and neurocognitive profiles (PRI, WMI, or PSI). It is of interest that CD62L and total MPs count appeared to be significantly associated only with PRI and not with WMI or PSI performances.

### 3.6. Multimodal Study for Cerebral White Matter Integrity among the Study Subjects

Three bilateral white matter tracts (bilateral anterior corona radiata, ACR; superior corona radiata, SCR; and superior longitudinal fasciculus, SLF) were chosen based on the prevalence of WMHs (location-wise), as seen in the MRI brain images of the study subjects. Therefore, six ROIs were selected, regarding the presence of WMHs in specific tracts: right ACR (RACR) (*n* = 7), left ACR (LACR) (*n* = 7), right SCR (RSCR) (*n* = 4), left SCR (LSCR) (*n* = 4), right SLF (RSLF) (*n* = 5), and left SLF (LSLF) (*n* = 3) (see Figure 1). White matter integrity was defined by the value of diffusion parameters of certain white matter tracts, such as (1) fractional anisotropy (FA), where lower FA signifies reduced white matter integrity due to reduced restriction in molecular motion following disruption in axonal cell membranes and myelin sheathes [36,37]; (2) mean diffusivity (MD), where increased MD also signifies reduced white matter integrity following an increase in mean molecular motion concerning disruption in cellular properties (i.e., integrity and size) [37,38]; and (3) radial diffusivity (RD) and axial diffusivity (AD), defined by the rate of diffusion along cellular axes [39], where RD/AD varies across brain regions [40].

We found that, compared with WMH^−^ subjects, WMH^+^ subjects had slightly lower FA, higher MD, and various ADs/RDs in all selected tracts. However, as shown in Table 4, the differences were not significant. Furthermore, FA had a significant negative correlation (low to moderate) with age and QRISK2, whereas AD had a significant negative correlation (low) with age only (Table 5). Further analysis, however, showed no association between FA, MD, AD, and RD with age and QRISK2. Hence, advancing age and percentage of QRISK2 do not seem to serve as direct measures of white matter integrity for the selected tracts in the present study. However, the linear model revealed a significant association between QRISK2 and the FA, MD, AD, and RD parameters of the LSLF tract. Hence, it can be postulated that a higher QRISK2 can predict a reduction in LSLF integrity (lower FA and higher MD; see Table 5).

Furthermore, there was a significant positive correlation of FA in the majority of selected white matter tracts, with PRI, WMI, and PSI, despite no significant association being found (Table 5). MD and RD were not correlated with any of the cognitive indices. Meanwhile, AD was positively correlated with PRI but not with WMI and PSI, despite no significant association being found (Table 5). Hence, the scores obtained from the three cognitive indices used in the present study did not seem to be correlated with the integrity of the selected white matter tracts.

No significant correlations were found between the diffusion parameters and MP counts. However, CD62L was positively correlated with the RD of bilateral ACR, bilateral SCR, and RSLF (not LSLF), although no linear relationship was found between CD62L and RD. It is of interest that we observed that the FA, MD, AD, and RD of LSLF had significant linear relationships with CD62L, CD146, and total MP. Hence, although the parameters were not always correlated, there is enough evidence to suggest that the enumeration of CD62L, CD146, and total MP count are associated, which may be of potential use to predict the white matter integrity of the LSLF tract.

## 4. Discussion

### 4.1. Cardiocerebrovascular Risk Prediction and Age as Surrogate Markers of CSVD

We found that WMH^+^ subjects had higher mean QRISK2 percentage scores. Moreover, older subjects with WMHs had a significantly greater risk than younger subjects with WMHs. Thus, WMH^+^ subjects seem likely to show a higher risk for developing cardiocerebrovascular disease. A previous study identified aging and hypertension as major cardiovascular risk factors for the development of WMHs [40]. It has been further supported that cardiovascular risk factors and aging are the main contributors to the prevalence of WMHs and increased WMHs volume in healthy adults [41]. It has been recognized that up to 95% of elderly individuals with greater cardiovascular risk have WMHs [42], although our data comprised relatively younger adults. Furthermore, numerous studies have shown an independent association between subclinical cardiovascular risk (i.e., diabetes and hypertension) with cerebral white matter microstructure in healthy middle-to-elderly aged population [43,44,45].

Although most of the previous research has found a significant association between age, cardiovascular risk, and WMHs, these were derived from studies involving Caucasian and Afro-Caribbean populations. To date, studies on Asian populations, specifically on WMHs, remain scarce. Previous studies have reported that Asian populations (i.e., among Malays and Chinese Singaporeans) had a higher burden in cardiocerebrovascular disease, with a higher percentage of risk factors [46,47] than those reported among Caucasians [48] and, hence, a higher prevalence of WMHs [49]. Recent studies involving other Asian populations (i.e., in Singapore, Korea, and Hong Kong) have also reported aging as contributing to higher cardiovascular burden, leading to an increased prevalence of WMHs in neuroimaging [46]. It is also alarming to note the likely adverse impacts of CSVD on the quality of life and longevity among growing aging populations in Asia.

We also found that increased QRISK2 scores were significantly associated with reduced white matter integrity (in terms of lower FA and higher MD) in the LSLF tract. However, many studies have already reported that vascular risk factors, such as hypertension, smoking, and lifestyle, are associated with white matter diffusion parameters [50,51,52,53]. Although the sensitivity of diffusion parameters in detecting white matter integrity has been widely described, their complex associations with modifiable vascular risk factors remain elusive [54]. However, the association of cardiovascular risk with the integrity of specific tracts is worth considering, as the prevalence of WMHs might only affect certain tracts. From this perspective, one study has recently reported that lower FA measures in specific tracts related to WMHs in healthy middle-aged adults have a statistically significant association with the presence of multiple vascular risk factors [55]. Collaboratively, our findings are consistent with those of others that argued that a decline in SLF white matter integrity has associations with uncontrolled vascular risk factors [56,57]. Changes in diffusion parameters in SLF have also been found to be associated with increased QRISK2; for instance, increased RD in the SLF has also been reported in subjects with dyslipidemia [57], without any report on bilateral involvements. Hence, our findings may infer that changes in the integrity of SLF tracts regarding cardiocerebrovascular risk appear to be more dominant in the left hemisphere (i.e., LSLF). In addition, the QRISK2 percentage score may serve as a practical parallel tool to gauge the integrity of SLF white matter tracts—at least, in the setting of asymptomatic subjects, for which occult CSVD may incidentally be detected in neuroimaging.

Furthermore, no significant relationship was found between age and FA, although the results suggested reduced SLF white matter integrity in older subjects. Hence, we postulate that advancing age may only negatively impact a portion of SLF. For instance, a previous study has shown that aging selectively impacts frontal white matter regions [58,59,60]. Several DTI studies have supported this “frontal aging hypothesis” and showed an anterior to posterior gradient in the decline of white matter integrity, with reduced FA and increased MD of frontal lobe structures despite the relative preservation of posterior brain regions [61,62,63,64,65].

In a routine clinical setting, our study supports the notion that an increased cardiocerebrovascular risk (as predicted by QRISK2 score) may serve as an objective and diagnostic guide to inform clinicians about the potential risk of underlying WMHs, specifically with respect to the CSVD spectrum in asymptomatic, economically productive young adults.

### 4.2. Neurocognitive Profiles as Surrogate Markers to CSVD

It has been reported that WMH^+^ subjects with vascular risk factors are more vulnerable to developing cognitive impairment [66]. Throughout the present study, only PSI was consistently correlated and associated with age and not with PRI and WMI. The variability of results between WAIS-IV indices used in the present study may be due to the small sample size and lack of follow-up. In addition, we recruited asymptomatic subjects as young as 25 years old. One study reported that, among 142 subjects, those aged 20–59 years (*n* = 95) had WMHs burden, which was not significantly associated with cognitive deficits, whereas participants aged 60 and older (*n* = 47) accounted for more than 58% of the variance in working memory, processing speed, fluency, and fluid intelligence performances [67].

The Ohasama Study [68] reported among a population of healthy Japanese adults (*n* = 331, age ≥ 60 years) with a 7 year follow-up that reductions in processing speed, perceptual reasoning, and memory appeared to be associated with silent cerebrovascular white matter lesions or WMHs. Similar findings have also been reported with respect to the association of the presence of WMHs with frontal executive function, memory, and global functioning [69]. By contrast, Sachdev et al. (2017) [70] did not find any significant association between the prevalence of WMHs with cognitive functions, especially on episodic memory, working memory, and general intellectual functioning in 478 subjects aged 60–64 years.

Furthermore, in the present study, we found a significant relationship with processing speed on the basis of PSI and age (but not QRISK2), where PSI was lower in WMH^+^ subjects. This finding was supported by the fact that the progression of WMHs in periventricular spaces has been recognized to affect cognitive impairment, particularly in terms of information processing speed and long-term functional decline [71]. However, both studies involved a population-based sample of non-demented and stroke-free individuals ranging between 60 and 90 years old, with a bigger sample size (sample size up to 1000). Finally, WMHs have also been previously reported to predict cognitive impairments in the elderly (age ≥ 60 years) with depression but were not associated with cognitive performance in healthy controls [72]. The total impact of WMHs burden has been reported to be minimal and unrelated to reduced cognitive performance among young-to-middle-aged adults (20–59 years old); however, WMHs have been frequently associated with low cognitive test performance among adults age 60 years and older [73].

We found no association between neurocognitive profiles and diffusion parameters, although a significant correlation was found between the profiles with FA of selected white matter tracts. According to Tuladhar et al., reduced white matter tract integrity in certain locations of white matter increased the likelihood of decline in specific cognitive functions [74]. Similarly, D’Souza et al. showed that changes of FA in white matter tracts significantly affected the performance of subjects in terms of cognitive function tests [75]. The Radboud University Nijmegen Diffusion Tensor and Magnetic Resonance Cohort (RUN DMC) cohort [76] initially studied 499 subjects with a prevalence of WMHs using WAIS digit span evaluations and found that FA was associated with attention and processing speed. The subsequent work of this cohort further demonstrated reduced FA and higher MD in the white matter tracts of symptomatic CSVD subjects (*n* = 115) compared with asymptomatic controls (*n* = 50), insinuating an association of white matter integrity with processing speed and executive function [77]. A similar observation has been reported in the Discontinuation of Antihypertensive Treatment in Elderly People (DANTE) study in Leiden [78], regarding cognitive and memory functions in healthy adults. Thus, the findings in the present study support the growing evidence indicating that white matter integrity, in terms of FA, can potentially be used as a neuroimaging marker for cognitive and memory ability in asymptomatic individuals, with or without the evidence of WMHs.

### 4.3. Microparticles as Surrogate Markers for CSVD

To the best of our knowledge, this research is the first to describe and show an elevated MPs counts (mean percentage) in asymptomatic WMH^+^ subjects, concerning select MP subpopulations. We found that platelets-derived MPs (PDMPs) (CD62P), leukocyte-derived MPs (LMPs) (CD62L), and total MPs count were significantly higher among WMH^+^ subjects. This may suggest the potential roles of certain MPs subpopulations (i.e., PDMPs and LMPs) in the prevalence of WMHs among our asymptomatic healthy subjects as well as the contribution of different MPs subpopulations to the total MPs count.

However, there is a lack in the literature regarding changes associated with the level of circulating MPs in asymptomatic individuals. To date, most studies have compared differences in the levels of MPs between healthy controls and symptomatic patients in vascular-related diseases. A higher level of LMPs in ischemic cerebrovascular disease patients compared with normal controls has been reported [79]. In addition, an increment in the level of endothelial cell-derived MPs (EMPs) (CD146) has also been found in CSVD symptomatic patients compared with healthy controls [15], thus depicting that EMPs may contribute to the progression of CSVD. Furthermore, several studies have found increases in the level of CD62P in symptomatic, lacunar stroke patients [78,80] only. Thus, elaborating the role of MPs levels in asymptomatic individuals may contribute to predicting future symptomatic events, if the MPs can be affirmatively determined as surrogate blood biomarkers for the spectrum of CSVD manifestations, including “silent” WMHs.

**Figure 2:**
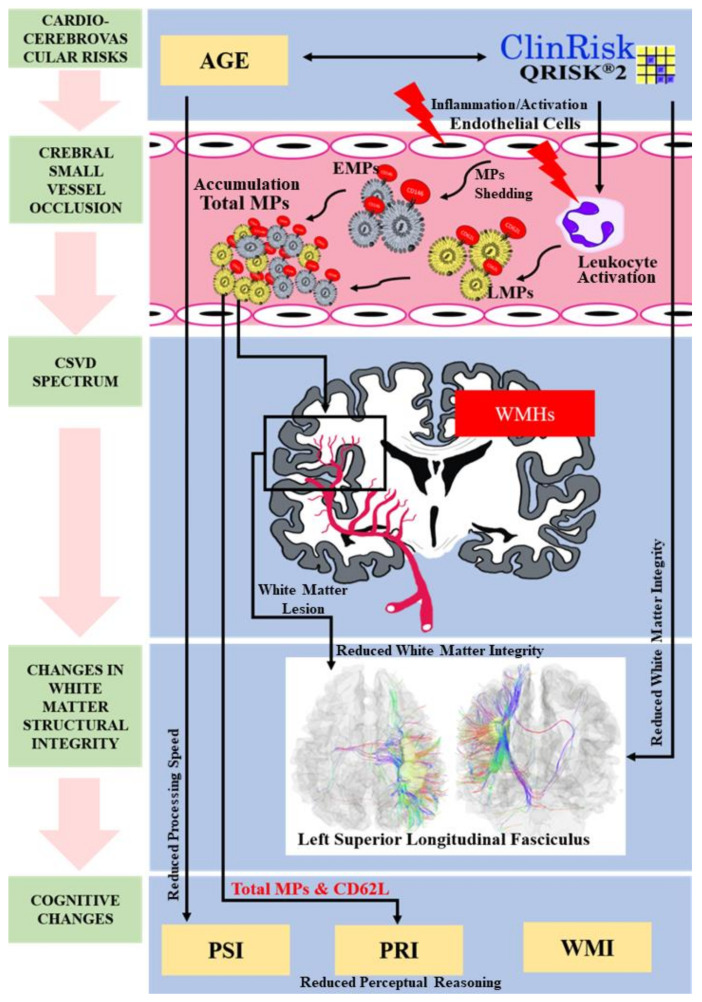
Multimodal linear relationship between surrogate markers: cardiocerebrovascular risk factors, such as age, are associated with QRISK2 and Wechsler Adult Intelligence Scale: Fourth edition (WAIS-IV) processing speed index (PSI). QRISK2 is associated with the leukocyte-derived MPs (LMPs; i.e., CD62L), whereas endothelial cell-derived MPs (EMPs; i.e., CD146) may exclusively be induced by other factors, inclusive of cardiocerebrovascular risk factors. LMPs and EMPs can potentially contribute to total MPs; hence, MP accumulation in cerebral small vessels (or end arteries) is thus postulated to cause occlusion. Such an occlusion may induce the formation of WMHs. LMPs, EMPs, and total MPs were associated with the integrity of the left superior longitudinal fasciculus (LSLF) regarding the presence of WMHs in that particular region. QRISK2 was also directly associated with the integrity of LSLF. Finally, total MPs and CD62L were exclusively associated with the WAIS-IV perceptual reasoning index (PRI).

PDMPs are a major contributor to microthrombi [81], supporting the findings of the present study, as PDMPs had the highest counts in WMH^+^ subjects. A cross-sectional study among 190 healthy Japanese men with predicted 10-year cardiocerebrovascular risk found that higher PDMPs were associated with increased cardiovascular diseases [82]. In another study, PDMPs were assumed to play a significant role in cardiovascular disease, serving as a major player in coagulation and the formation of microthrombi in blood vessels [83]. PDMPs have also been significantly associated with lesion volume in acute ischemic stroke but not in asymptomatic subjects with WMHs lesions on neuroimaging [84]. It is of interest that we found only LMPs (CD62L) to be associated with 10-year cardiocerebrovascular risk (QRISK2), rather than PDMPs, and to also not be associated with age and QRISK2 (see Figure 2).

Higher circulating LMPs have been demonstrated in patients with ischemic cerebrovascular disease [79], where healthy controls had a relatively low count compared with those who were symptomatic. As described in previous studies, greater counts of LMPs have been found in patients with recent cardiovascular events, thus inferring that an elevated level of circulating MPs reflects the underlying vessel occlusion in the form of microthrombi in symptomatic patients [85,86]. An increased risk of thrombosis has also been reported, concerning elevated LMPs levels in cardiovascular risk patients, when compared with healthy controls [87]. Thus, collectively, it seems that increased circulating MPs subpopulations (i.e., PDMPs and/or LMPs) and combinations of multiple MPs subpopulations (i.e., total MPs) have a potential predictive impact on the prevalence and progression of WMHs in CSVD among asymptomatic individuals (and, in the present study, among those aged 25–62 years; see Figure 2). Further research is required to confirm these findings, preferably with a follow-up study and collective population data.

In the present study, circulating MPs were featured as novel surrogate markers for the white matter integrity in CVSD, through the presence/absence of WMHs. However, no significant correlation was found between MPs and the diffusion parameters of the selected tracts. Nevertheless, significant associations were found between LSLF diffusion parameters and CD62L, CD146, and total MPs. A plausible reason for this attracts an argument based on a previous study, which opined that increased FA in certain tracts might also be due to decreases in axonal diameter and branching [36]. Essentially, it is presumed that the occluded small penetrating vessels inflict a subclinical ischemic insult in the LSLF region, which may potentially contribute to the decline in white matter integrity in that specific tract over time unless perfusion is restored. However, the exact molecular and cellular mechanisms underlying the changes in diffusion parameters (i.e., FA) remain unknown [88]. Hence, careful interpretation on the changes in FA is necessary, as increased FA can also be due to neuronal degeneration, such as in crossing fibers [89].

Furthermore, changes in the AD parameter of the LSLF were found to correlate with higher circulating CD62L, CD146, and total MPs count. Previous studies (in both humans and animal) have indicated that the reduction of AD might be due to the effects of ischemia, thus suggesting that axonal degeneration (regarding ischemia) may lead to a subsequent reduction in diffusion coherence in terms of directionality (i.e., AD) [90,91]. These, too, appear to arise from aging, by which (as described before) AD seems to decrease with age, chiefly from age-related demyelination [92]. It is of interest that, in the present study, we also found that CD62L is associated with QRISK2 and the PRI cognitive index, whereas total MPs were exclusively associated with PRI performance. Hence, it can be speculated that the CD62L subpopulation within the micro-thrombus formation may play a crucial role in inducing ischemic demyelination in CSVD (see Figure 2) and, thus, may serve as a plausible biomarker for vascular-related cognitive impairment associated with CSVD.

### 4.4. Strengths and Limitations of the Study

In this study, we established multimodal associations as potential surrogate markers for “silent” CSVD manifestation in well-characterized cardiocerebrovascular demographics among a relatively young sample of neurologically asymptomatic adults. To the best of our knowledge, this study is the first to exhibit elevated MPs counts in asymptomatic CSVD, which warrants further delineation. However, the study was conducted in a single center clinical setting with small sample size (*n* = 60) in order to explore the prevalence of WMHs, thus limiting the generalizability of the study findings. Moreover, as part of the study inclusion/exclusion criteria, many older adults were excluded due to their high QRISK2 scores (more than 20%) and were regarded as symptomatic individuals. In addition, WAIS-IV is labor-intensive and client-demanding to complete, which better suits the research setting and is impractical for routine daily clinical use for neurocognitive screening. Nevertheless, we consolidated the relevant WAIS-IV indices for the purposes of this research in order to ensure comprehensiveness of the assessment. Finally, the candidate MPs selected and tested in this study represent a panel of MPs, selected among other MPs reported at the time of research commencement. Thus, extending the involvement of other/new MPs subpopulations in this context remains unknown.

## 5. Conclusions

The present study, to the best of our knowledge, was the first to examine the presence/absence of WMHs to depict CSVD in apparently asymptomatic individuals using three modalities, namely, neurocognitive, microparticles profiling, and neuroimaging (dMRI), in a selected cohort of subjects with variable cardiocerebrovascular risk (as determined by QRISK2 predictive score). Although it was carried out at a single center with a small sample size, the present study established “silent” CSVD manifestations in apparently asymptomatic individuals from well-characterized cardiovascular demographics in relatively young, neurologically asymptomatic adults from suburban southeast Peninsular Malaysia.

## Figures and Tables

**Table 1:** Distribution of demographics variables in the study (*N* = 60, mean age = 39.83 ± 11.50 years).

	Variables	*n* (%)
Age	<20	0 (0)
	21–40	31 (51.7)
	41–60	26 (43.3)
	>60	3 (5)
Gender	Male	19 (31.7)
	Female	41 (68.3)
Ethnic	Malay	54 (90.0)
	Chinese	4 (6.7)
	Other	2 (3.3)
WMHs *	Absent (WMH^−^)	40 (66.7)
	Present (WMH^+^)	20 (33.3)
Smoking status	Nonsmoker	52 (86.7)
	Ex-smoker	7 (11.7)
	Light smoker	1 (1.7)
Family history of coronary heart disease in 1st degree relative under 60 years old	Yes	15 (25.0)
	No	45 (75.0)
Treated Hypertension	Yes	9 (15.0)
	No	51 (85.0)
Atrial fibrillation	Yes	-
	No	60 (100)
Type 2 Diabetes	Yes	-
	No	60 (100)
Hyperlipidemia	Yes	-
	No	60 (100)
Chronic kidney disease (stage 4 or 5)	Yes	-
	No	60 (100)

* WMHs, white matter hyperintensities.

**Table 2:** Independent *T*-tests for comparison of the study variables between WMH^+^ and WMH^-^ subjects.

Variables	Mean ± SD		T-Statistics (*df*)	*p*-Value
	WMH^-^ (*n* = 20)	WMH^+^ (*n* = 40)		
Age	36.75 ± 10.04	46.00 ± 12.00	−2.98 (32.66)	0.006 *
QRISK2	1.32 ± 1.54	5.84 ± 6.43	−4.24 (58)	0.000 *
PRI	102.95 ± 13.30	101.30 ± 10.73	0.52 (46.12)	0.607
WMI	108.53 ± 19.14	105.25 ± 14.55	0.74 (48.45)	0.465
PSI	103.73 ± 14.31	98.05 ± 11.49	1.66 (46.33)	0.104
CD62L	98.30 ± 42.43	163.85 ± 140.72	−2.73 (58)	0.008 *
CD235a	33.05 ± 74.25	50.45 ± 42.10	−1.16 (56.95)	0.252
CD62P	150.45 ± 75.21	370.80 ± 330.271	−4.05 (58)	0.000 *
CD146	25.08 ± 20.27	194.80 ± 669.90	−1.62 (58)	0.112
Total MPs	306.88 ± 152.15	779.90 ± 930.04	−3.16 (58)	0.003 *

Notes: WMH, white matter hyperintensities; SD, standard deviation; df, degree of freedom; PRI, perceptual reasoning index; WMI, working memory index; PSI, processing speed index; CD, cluster differentiations; MPs, microparticles. * *p* = significant difference (1-tailed) at the 0.01 level.

**Table 3:** Correlation and linear regression profile of study variables.

No.	Variables	*r* (*β*)				
		1	2	3	4	5
1	Age	1				
2	QRISK2	0.75 * (0.57 *)	1			
3	PRI	−0.27 * (0.02)	−0.23 * (−0.09)	1		
4	WMI	−0.35 * (−0.01)	−0.32 * (−0.07)	0.62 * (0.35 *)	1	
5	PSI	−0.56 * (−0.31*)	−0.40 * (−0.19)	0.60 * (0.44 *)	0.61 * (0.63 *)	1
6	CD62L	0.51 * (0.19)	0.58 * (0.41 *)	0.09 (0.25 *)	−0.12 (0.12)	−0.19 (−0.18)
7	CD235a	0.26 * (0.18)	0.13 (0.02)	0.10 (0.11)	0.05 (0.19)	−0.16 (−0.15)
8	CD62P	0.32 * (0.16)	0.44 * (−0.08)	0.08 (0.32)	−0.15 (−0.09)	−0.15 (0.01)
9	CD146	0.26 * (0.14)	0.41 * (0.28)	−0.18 (−0.16)	−0.14 (−0.42)	−0.07 (0.05)
10	Total MPs	0.40 * (−0.02)	0.54 * (0.30)	−0.06 (−0.34 *)	−0.16 (−0.12)	−0.15 (0.00)

Notes: *r* = Pearson correlation coefficient, *β* = standardized beta coefficients; PRI, perceptual reasoning index; WMI, working memory index; PSI, processing speed index; CD, cluster differentiations; MPs, microparticles. * *p* = significant difference (1-tailed) at the 0.01 level.

**Table 4:** Comparison of the integrity of selected white matter between WMH^+^ and WMH^-^ subjects with independent *T*-tests.

Tracts Parameters	(Mean ± SD)		*T*-Statistics (*df*)	*p*-Value
	WMH^−^	WMH^+^		
RACR				
FA	0.42 ± 0.03	0.42 ± 0.03	0.35 (37.94)	0.727
MD	0.82 ± 0.07	0.84 ± 0.08	−0.59 (33.83)	0.560
AD	1.23 ± 0.13	1.24 ± 0.14	−0.34 (35.39)	0.733
RD	0.63 ± 0.05	0.64 ± 0.06	−0.86 (32.21)	0.395
LACR				
FA	0.42 ± 0.02	0.42 ± 0.27	0.45 (34.42)	0.649
MD	0.82 ± 0.08	0.83 ± 0.08	−0.24 (38.36)	0.812
AD	1.23 ± 0.13	1.23 ± 0.13	−0.05 (38.38)	0.957
RD	0.62 ± 0.05	0.63 ± 0.05	−0.46 (37.81)	0.645
RSCR				
FA	0.46 ± 0.03	0.46 ± 0.02	−0.06 (48.00)	0.955
MD	0.81 ± 0.07	0.81 ± 0.07	−0.05 (37.40)	0.958
AD	1.26 ± 0.13	1.26 ± 0.12	−0.02 (40.09)	0.987
RD	0.59 ± 0.04	0.59 ± 0.05	−0.09 (34.21)	0.923
LSCR				
FA	0.46 ± 0.02	0.46 ± 0.02	0.17 (41.85)	0.866
MD	0.82 ± 0.07	0.83 ± 0.08	−0.30 (34.89)	0.765
AD	1.26 ± 0.12	1.27 ± 0.13	−0.16 (36.26)	0.875
RD	0.59 ± 0.05	0.60 ± 0.06	−0.45 (33.64)	0.655
RSLF				
FA	0.40 ± 0.02	0.40 ± 0.03	0.68 (34.32)	0.499
MD	0.77 ± 0.07	0.78 ± 0.08	−0.24 (34.15)	0.813
AD	1.13 ± 0.11	1.14 ± 0.12	−0.00 (35.76)	0.998
RD	0.59 ± 0.05	0.60 ± 0.06	−0.49 (32.15)	0.627
LSLF				
FA	0.41 ± 0.02	0.41 ± 0.03	0.94 (34.30)	0.355
MD	0.78 ± 0.07	0.78 ± 0.08	−0.13 (33.43)	0.897
AD	1.15 ± 0.11	1.15 ± 0.12	0.14 (34.98)	0.892
RD	0.59 ± 0.05	0.60 ± 0.06	−0.41 (31.77)	0.683

Notes: AD, axial diffusivity; FA, fractional anisotropy; LACR, left anterior corona radiata; LSCR, left superior corona radiata; LSLF, left superior longitudinal fasciculus; MD, mean diffusivity; RACR, right anterior corona radiata; RD, radial diffusivity; RSCR, right superior corona radiata; RSLF, right superior longitudinal fasciculus; WMH, white matter hyperintensity, *p* = significant difference.

**Table 5:** Correlation and multiple linear regression profile of study variables with white matter integrity.

Variables & Tracts	*r* (*β*)			
	FA	MD	AD	RD
Age				
RACR	−0.43 * (1.23)	−0.13 (−0.78)	−0.23 * (−2.52)	0.00 (1.46)
LACR	−0.42 * (1.15)	−0.14 (−1.96)	−0.23 * (−2.44)	−0.01 (2.74)
RSCR	−0.38 * (0.17)	−0.11 (−0.53)	−0.20 * (0.43)	0.03 (0.16)
LSCR	−0.40 * (−0.80)	−0.13 (−0.26)	−0.21 * (0.74)	−0.02 (−1.26)
RSLF	−0.46 * (−0.32)	−0.14 (−0.85)	−0.23 * (−0.72)	−0.02 (0.27)
LSLF	−0.37 * (2.76)	−0.16 (1.18)	−0.24 * (−5.73)	−0.07 (4.81)
QRISK2				
RACR	−0.28 * (3.90)	0.05 (−0.67)	−0.04 (−7.32)	0.16 (4.84)
LACR	−0.26 * (−1.87)	0.05 (−1.41)	−0.03 (4.26)	0.15 (−2.42)
RSCR	−0.24 * (0.47)	0.05 (1.44)	−0.03 (−1.86)	0.16 (0.63)
LSCR	−0.30 * (−0.41)	0.05 (1.81)	−0.03 (0.69)	0.14 (−0.97)
RSLF	−0.35 * (−2.94)	0.07 (−0.75)	−0.02 (5.14)	0.18 (−3.47)
LSLF	−0.35 * (4.09 *)	0.06 (3.07 *)	−0.03 (−9.09 *)	0.17 (8.11 *)
PRI				
RACR	0.45 * (−3.55)	0.15 (−0.21)	0.24 * (8.21)	0.03 (−4.79)
LACR	0.45 * (1.69)	0.14 (0.67)	0.23 * (−3.15)	0.03 (1.75)
RSCR	0.33* (2.61)	0.14 (−0.07)	0.21 * (−7.64)	0.05 (4.25)
LSCR	0.39* (−1.03)	0.16 (0.76)	0.23 * (5.11)	0.07 (−2.65)
RSLF	0.45* (−0.06)	0.12 (0.41)	0.21 * (0.49)	0.02 (−1.47)
LSLF	0.42* (0.57)	0.16 (1.27)	0.24 * (−1.67)	0.07 (1.49)
WMI				
RACR	0.28* (−0.80)	0.03 (0.08)	0.09 (1.20)	−0.06 (0.30)
LACR	0.32* (−0.14)	0.01 (0.21)	0.08 (2.30)	−0.09 (−2.20)
RSCR	0.14 (−1.17)	0.00 (−0.92)	0.05 (0.44)	−0.06 (−0.17)
LSCR	0.25 * (1.95)	0.00 (0.14)	0.06 (−4.05)	−0.07 (3.13)
RSLF	0.36 * (−1.85)	−0.00 (−0.07)	0.08 (5.10)	−0.11 (−4.89)
LSLF	0.28 * (0.23)	0.00 (0.98)	0.07 (−0.27)	−0.06 (0.59)
PSI				
RACR	0.28 * (0.49)	−0.04 (−0.79)	0.04 (−0.79)	−0.14 (1.47)
LACR	0.27 * (−1.68)	−0.05 (0.82)	0.03 (3.85)	−0.15 (−3.95)
RSCR	0.20 * (−0.73)	−0.06 (0.51)	0.01 (0.35)	−0.15 (0.24)
LSCR	0.31 * (2.10)	−0.06 (0.90)	0.02 (−4.71)	−0.16 (3.25)
RSLF	0.35 * (−0.14)	−0.07 (0.54)	0.02 (0.81)	−0.18 (−1.22)
LSLF	0.33 * (−2.02)	−0.03 (0.99)	0.05 (6.11)	−0.13 (−4.10)
CD62L				
RACR	−0.03 (1.36)	0.17 (−0.70)	0.13 (−1.48)	0.21 * (1.05)
LACR	−0.04 (−1.08)	0.16 (−0.89)	0.12 (1.42)	0.21 * (−0.49)
RSCR	−0.03 (−0.02)	0.17 (0.07)	0.13 (−0.46)	0.22 * (0.04)
LSCR	−0.05 (−0.19)	0.17 (0.57)	0.14 (1.15)	0.21 * (−0.69)
RSLF	−0.08 (−2.96)	0.16 (0.36)	0.11 (5.48)	0.21 * (−4.42)
LSLF	−0.03 (5.05 *)	0.13 (2.28 *)	0.10 (−11.61 *)	0.16 (9.45 *)
CD235a				
RACR	0.00 (−4.68)	0.00 (−0.79)	−0.00 (7.79)	0.00 (−5.04)
LACR	0.03 (2.40)	0.00 (1.31)	0.00 (−2.55)	0.00 (2.43)
RSCR	0.00 (−1.24)	0.04 (1.05)	0.03 (4.03)	0.06 (−1.63)
LSCR	0.07 (1.99)	0.01 (0.73)	0.02 (−5.03)	0.01 (3.15)
RSLF	−0.07 (2.49)	0.00 (−0.45)	−0.02 (−7.30)	0.03 (4.12)
LSLF	0.06 (1.15)	−0.04 (1.05)	−0.03 (−2.73)	−0.05 (2.07)
CD62P				
RACR	0.10 (1.99)	0.11 (−0.06)	0.11 (−3.68)	0.11 (2.82)
LACR	0.09 (−1.42)	0.11 (1.46)	0.10 (4.12)	0.11 (−2.47)
RSCR	0.10 (−1.23)	0.08 (0.92)	0.09 (1.36)	0.07 (−1.98)
LSCR	0.09 (2.23)	0.14 (−0.40)	0.13 (−3.38)	0.15 (3.99)
RSLF	−0.03 (−2.35)	0.08 (−0.01)	0.06 (2.97)	0.12 (−3.12)
LSLF	0.04 (3.61)	0.07 (2.02)	0.06 (−8.37)	0.08 (6.87)
CD146				
RACR	0.02 (2.12)	0.02 (0.77)	0.02 (−5.13)	0.02 (2.89)
LACR	0.06 (−2.21)	0.03 (1.26)	0.03 (6.05)	0.02 (−3.16)
RSCR	−0.00 (2.29)	0.02 (1.91)	0.02 (−6.25)	0.03 (2.79)
LSCR	0.01 (−1.95)	0.00 (0.12)	0.00 (5.04)	0.00 (−3.31)
RSLF	0.0 (−3.16)	0.06 (−0.98)	0.05 (8.12)	0.06 (−4.57)
LSLF	0.03 (5.06 *)	−0.02 (2.26 *)	−0.02 (−12.01 *)	−0.03 (8.50 *)
Total MPs				
RACR	0.07 (2.31)	0.09 (0.07)	0.08 (−5.08)	0.09 (3.26)
LACR	0.08 (−2.00)	0.10 (1.91)	0.09 (5.99)	0.10 (−3.33)
RSCR	0.06 (−0.53)	0.08 (1.50)	0.08 (0.18)	0.08 (−1.11)
LSCR	0.04 (1.09)	0.12 (−0.09)	0.10 (−1,67)	0.13 (2.23)
RSLF	−0.04 (−2.66)	0.09 (−0.29)	0.06 (4.45)	0.12 (−3.43)
LSLF	0.04 (4.82 *)	0.06 (2.77 *)	0.04 (−11.17 *)	0.07 (8.71 *)

Notes: *r* = Pearson correlation coefficient; *β* = standardized beta coefficients; * *p* = significant difference (1-tailed) at the 0.01; AD, axial diffusivity; CD, cluster differentiation; FA, fractional anisotropy; LACR, left anterior corona radiata; LSCR, left superior corona radiata; LSLF, left superior longitudinal fasciculus; MD, mean diffusivity; PRI, perceptual reasoning index; PSI, processing speed index; RACR, right anterior corona radiata; RD, radial diffusivity; RSCR, right superior corona radiata; RSLF, right superior longitudinal fasciculus; WMI, working memory index.

## Data Availability

We declare that the materials described in the manuscript, including all relevant raw data, will be freely available to any scientist wishing to use them for noncommercial purposes, without breaching participant confidentiality.
